# 9-Amino­acridin-10-ium 4-amino­benzo­ate dihydrate

**DOI:** 10.1107/S160053681401023X

**Published:** 2014-05-17

**Authors:** Nallathambi Dhanabalan, Kaliyaperumal Thanigaimani, Suhana Arshad, Ibrahim Abdul Razak, K. Joseph Santhanaraj

**Affiliations:** a Department of Chemistry, Roever College of Engineering and Technology, Elambalur, Perambalur 621 212, Tamil Nadu, India; b School of Physics, Universiti Sains Malaysia, 11800 USM, Penang, Malaysia; c Department of Chemistry, St.Josephs College, Tiruchirappalli 620 002, Tamil Nadu, India

## Abstract

The asymmetric unit of the title hydrated salt, C_13_H_11_N_2_^+^·C_7_H_6_NO_2_^−^·2H_2_O, consists of two independent 9-amino­acridinium cations, two 4-amino­benzoate anions and four water mol­ecules. Both 9-amino­acridinium cations are essentially planar, with maximum deviations of 0.034 (1) and 0.025 (2) Å, and are protonated at the pyridine N atoms. The 4-amino­benzoate anions are approximately planar, with dihedral angles of 9.16 (19) and 5.4 (2)° between the benzene ring and the carboxyl­ate group. In the crystal, the two independent anions are connected by N—H⋯O hydrogen bonds, forming a layer parallel to (100). The layers are connected through the cations by N—H⋯N and N—H⋯O hydrogen bonds. The water mol­ecules, which form O—H⋯O hydrogen-bonded chains along the *b-*axis direction, connect the anions and the cations by O—H⋯O, N—H⋯O and C—H⋯O hydrogen bonds. The crystal structure also features π–π inter­actions [centroid–centroid distances = 3.6343 (9)–3.8366 (10) Å] and a C—H⋯π inter­action.

## Related literature   

For background to and the biological activity of acridine derivatives, see: Shubber *et al.* (1986 ▶); Sondhi *et al.* (2006 ▶); Salamanca & Khalil (2005 ▶). For related structures, see: Aghabozorg *et al.* (2010 ▶); Mei & Wolf (2004 ▶). For bond-length data, see: Allen *et al.* (1987 ▶). For stability of the temperature controller used for data collection, see: Cosier & Glazer (1986 ▶).
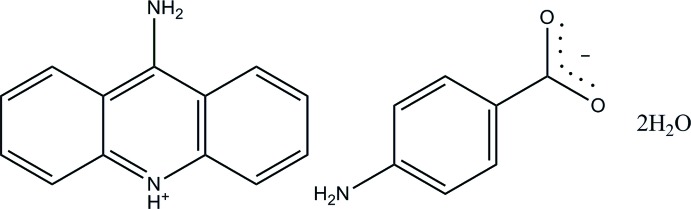


## Experimental   

### 

#### Crystal data  

C_13_H_11_N_2_^+^·C_7_H_6_NO_2_^−^·2H_2_O
*M* *_r_* = 367.40
Monoclinic, 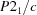

*a* = 25.6891 (9) Å
*b* = 7.2800 (2) Å
*c* = 21.6485 (6) Å
β = 114.865 (1)°
*V* = 3673.32 (19) Å^3^
*Z* = 8
Mo *K*α radiation
μ = 0.09 mm^−1^
*T* = 100 K
0.64 × 0.14 × 0.11 mm

#### Data collection  

Bruker SMART APEXII CCD area-detector diffractometer
Absorption correction: multi-scan (*SADABS*; Bruker, 2009 ▶) *T*_min_ = 0.942, *T*_max_ = 0.990
39382 measured reflections
10114 independent reflections
7214 reflections with *I* > 2σ(*I*)
*R*_int_ = 0.047

#### Refinement  

*R*[*F* ^2^ > 2σ(*F* ^2^)] = 0.052
*wR*(*F* ^2^) = 0.128
*S* = 1.04
10114 reflections
559 parameters
1 restraint
H atoms treated by a mixture of independent and constrained refinement
Δρ_max_ = 0.53 e Å^−3^
Δρ_min_ = −0.25 e Å^−3^

### 

Data collection: *APEX2* (Bruker, 2009 ▶); cell refinement: *SAINT* (Bruker, 2009 ▶); data reduction: *SAINT*; program(s) used to solve structure: *SHELXTL* (Sheldrick, 2008 ▶); program(s) used to refine structure: *SHELXTL*; molecular graphics: *SHELXTL*; software used to prepare material for publication: *SHELXTL* and *PLATON* (Spek, 2009 ▶).

## Supplementary Material

Crystal structure: contains datablock(s) global, I. DOI: 10.1107/S160053681401023X/is5353sup1.cif

Structure factors: contains datablock(s) I. DOI: 10.1107/S160053681401023X/is5353Isup2.hkl

Supporting information file. DOI: 10.1107/S160053681401023X/is5353Isup3.cml

CCDC reference: 1001221

Additional supporting information:  crystallographic information; 3D view; checkCIF report

## Figures and Tables

**Table 1:** Hydrogen-bond geometry (Å, °) *Cg*13 is the centroid of the C1*A*–C6*A* ring.

*D*—H⋯*A*	*D*—H	H⋯*A*	*D*⋯*A*	*D*—H⋯*A*
O1*W*—H1*W*1⋯O3*W*	0.84 (3)	1.92 (3)	2.758 (2)	179 (5)
O1*W*—H2*W*1⋯O1*B*	0.86 (3)	1.90 (3)	2.767 (2)	177 (2)
O2*W*—H1*W*2⋯O2*A* ^i^	0.88 (3)	1.92 (3)	2.8066 (19)	176 (3)
O3*W*—H2*W*3⋯O1*W* ^i^	0.96 (3)	1.72 (3)	2.676 (2)	174 (2)
O3*W*—H1*W*3⋯O2*B* ^ii^	0.90 (3)	1.79 (3)	2.674 (2)	166 (3)
O4*W*—H2*W*4⋯O1*A*	0.87 (3)	1.84 (3)	2.688 (2)	164 (3)
N1*A*—H1*NA*⋯O2*B* ^i^	0.92 (2)	2.09 (2)	2.946 (2)	156.0 (19)
N1*A*—H2*NA*⋯O1*B* ^iii^	0.92 (3)	2.12 (3)	2.983 (2)	158 (2)
N2*A*—H3*NA*⋯O2*A* ^i^	0.90 (2)	1.98 (2)	2.882 (2)	173 (2)
N2*B*—H3*NB*⋯O1*B* ^iv^	0.98 (2)	1.93 (2)	2.8695 (18)	160 (2)
N3*A*—H4*NA*⋯O4*W* ^v^	0.93 (2)	1.93 (2)	2.8227 (19)	161.6 (18)
N3*A*—H5*NA*⋯N1*B* ^vi^	0.91 (2)	2.20 (2)	3.031 (2)	151.4 (18)
N3*B*—H5*NB*⋯O3*W*	0.90 (2)	1.94 (2)	2.819 (2)	165 (2)
N3*B*—H4*NB*⋯N1*A* ^i^	1.00 (3)	2.16 (2)	3.067 (2)	151.3 (19)
C12*A*—H12*A*⋯O4*W* ^v^	0.95	2.55	3.4756 (19)	164
C16*B*—H16*B*⋯O3*W*	0.95	2.51	3.438 (2)	164
C11*A*—H11*A*⋯*Cg*13^iv^	0.95	2.95	3.6659 (19)	133

Symmetry codes: (i) 
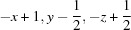
; (ii) 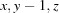; (iii) 
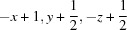
; (iv) 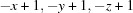; (v) 
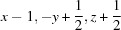
; (vi) 
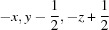
.

**Fig. 1.**
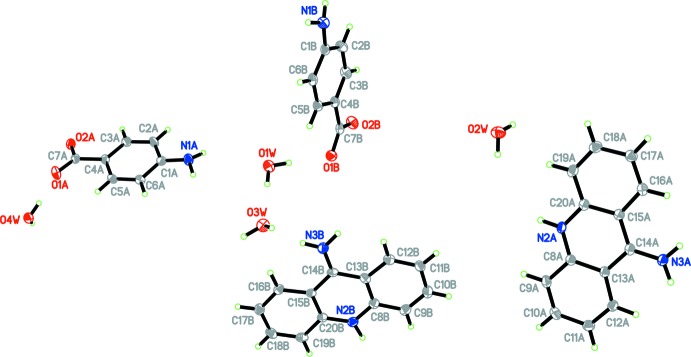
The molecular structure of the title compound with atom labels with 50% probability displacement ellipsoids.

**Fig. 2.**
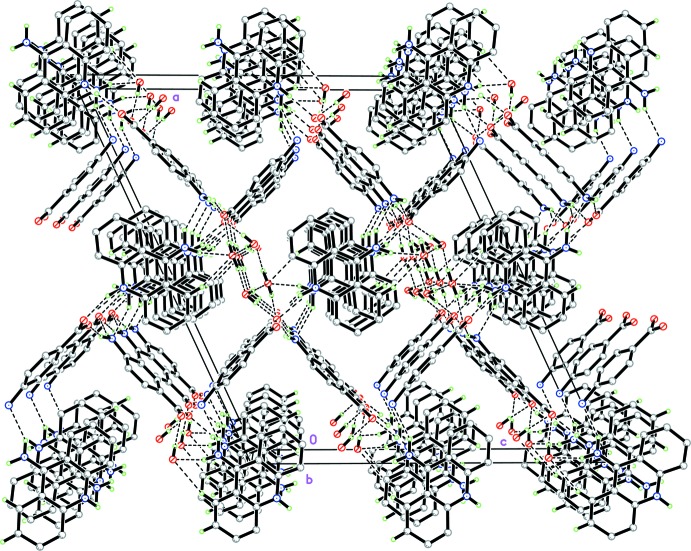
The crystal packing of the title compound. The H atoms not involved in the intermolecular interactions (dashed lines) have been omitted for clarity.

## supplementary crystallographic information

### 1. Comment

9-Aminoacridine (9-AA) and its derivatives present a very interesting object of research. This group of compounds exhibits a wide spectrum of biological activities: antibacterial, mutagenic, antitumor and antiinflammatory (Shubber *et al.*, 1986; Sondhi *et al.*, 2006; Salamanca & Khalil, 2005) In the viewpoint of crystal engineering, acridine and its 9-amino derivative are very interesting because of their capability for hydrogen bonding *via* N atom of the ring and π–π stacking since they possess three rings (Aghabozorg *et al.*, 2010; Mei & Wolf, 2004). In order to study potential hydrogen bonding interactions the crystal structure determination of the title compound (I) was carried out.

The asymmetric unit of the title salt contains two 9-aminoacridinium cations (*A* and *B*), two 4-aminobenzoate anions (*A* and *B*) and four water molecules (Fig. 1). Each 9-aminoaciridinium cation is essentially planar, with a maximum deviation of 0.034 (1) Å for atom C9A (molecule *A*) and 0.025 (2) Å for atom C11B (molecule *B*). The protonation of atoms N2A and N2B lead to a slight increase in C8A—N2A—C20A [122.22 (14)°] and C8B—N2B—C20B [121.86 (14)°] angles. The bond lengths (Allen *et al.*, 1987) and angles are normal.

In the crystal packing (Fig. 2), the cations and anions are linked by N1A—H1NA···O2B^i^, N1A—H2NA···01B^ii^, N3A—H5NA···N1B^iv^, N3B—H4NB···N1A^i^, N2A—H3NA···O2A^i^ and N2B—H3NB···O1B^v^ hydrogen bonds (symmetry code in Table 1). In addition, the water molecules are connected *via* O1W—H1W1···O3W and O3W—H2W3···O1W^i^ hydrogen bonds to form a one-dimensional supramolecular chain along the *b*-axis. Furthermore, the chains formed by water molecules and the ions [cations (A & B) and anions (A & B)] are connected *via* O1W—H2W1···O1B, O4W—H2W4···O1A, N3B—H5NB···O3W, N3A—H4NA···O4W^iii^, O2W—H1W2···O2A^i^, O3W—H1W3···O2B^vi^, C16A—H16B···O3W and C12A—H12A···O4W^iii^ hydrogen bonds (symmetry code in Table 1), forming a three-dimensional supramolecular network. Further, the cations (*A* & *B*) can establish several π–π interactions, with centroid···centroid distances in the range from 3.6343 (9) to 3.8366 (10) Å (C*g*1–C*g*3 = 3.6343 (9) Å, C*g*1=N2A/C8A/C20A/C13A–C15A and C*g*3 = C15A–C20A; C*g*1–C*g*1 = 3.7520 (9) Å; C*g*2–C*g*3 = 3.7822 (10) Å, *Cg*2 = C8A–C13A; C*g*7–C*g*7 = 3.7441 (9) Å, *Cg*7 = N2B/C8B/C20B/C13B–C15B; C*g*7–C*g*9 = 3.7434 (10) Å, C*g*9 = C15B–C20B; C*g*8–C*g*9 = 3.8193 (10) Å, C*g*8 = C8B–C13B; C*g*7–C*g*8 = 3.8366 (10) Å) and C—H···π interactions (Table 1) involving the C1A–C6A (centroid C*g*13) ring.

### 2. Experimental

Hot methanol solutions (20 ml) of 9-aminoacridine (48 mg, Aldrich) and 4-aminobenzoic acid (34 mg, Loba) was warmed for a half an hour over a water bath. The resulting solution was allowed to cool slowly at room temperature and crystals of the title compound (I) appeared after a few days.

### 3. Refinement

O- and N-bound H atoms were located in a difference Fourier maps and were refined freely, except for atom H5NB which was refined with a bond length restraint N—H = 0.87 (1) Å [refined distances: O—H = 0.84 (3)–0.96 (3) Å and N—H = 0.86 (2)–0.99 (2) Å]. The remaining H atoms were positioned geometrically (C—H = 0.95 Å) and refined using a riding model, with *U*_iso_(H) = 1.2*U*_eq_(C).

### Crystal data

**Table:**

C_13_H_11_N_2_^+^·C_7_H_6_NO_2_^−^·2H_2_O	*F*(000) = 1552
*M**_r_* = 367.40	*D*_x_ = 1.329 Mg m^−^^3^
Monoclinic, *P*2_1_/*c*	Mo *K*α radiation, λ = 0.71073 Å
Hall symbol: -P 2ybc	Cell parameters from 9979 reflections
*a* = 25.6891 (9) Å	θ = 2.6–30.0°
*b* = 7.2800 (2) Å	µ = 0.09 mm^−^^1^
*c* = 21.6485 (6) Å	*T* = 100 K
β = 114.865 (1)°	Needle, yellow
*V* = 3673.32 (19) Å^3^	0.64 × 0.14 × 0.11 mm
*Z* = 8	

### Data collection

**Table:**

Bruker SMART APEXII CCD area-detector diffractometer	10114 independent reflections
Radiation source: fine-focus sealed tube	7214 reflections with *I* > 2σ(*I*)
Graphite monochromator	*R*_int_ = 0.047
φ and ω scans	θ_max_ = 29.5°, θ_min_ = 2.1°
Absorption correction: multi-scan (*SADABS*; Bruker, 2009)	*h* = −34→35
*T*_min_ = 0.942, *T*_max_ = 0.990	*k* = −10→10
39382 measured reflections	*l* = −29→28

### Refinement

**Table:**

Refinement on *F*^2^	Primary atom site location: structure-invariant direct methods
Least-squares matrix: full	Secondary atom site location: difference Fourier map
*R*[*F*^2^ > 2σ(*F*^2^)] = 0.052	Hydrogen site location: inferred from neighbouring sites
*wR*(*F*^2^) = 0.128	H atoms treated by a mixture of independent and constrained refinement
*S* = 1.04	*w* = 1/[σ^2^(*F*_o_^2^) + (0.0477*P*)^2^ + 1.9401*P*] where *P* = (*F*_o_^2^ + 2*F*_c_^2^)/3
10114 reflections	(Δ/σ)_max_ = 0.001
559 parameters	Δρ_max_ = 0.53 e Å^−^^3^
1 restraint	Δρ_min_ = −0.25 e Å^−^^3^

### Special details

**Table:**

Experimental. The crystal was placed in the cold stream of an Oxford Cryosystems Cobra open-flow nitrogen cryostat (Cosier & Glazer, 1986) operating at 100.0 (1) K.
Geometry. All e.s.d.'s (except the e.s.d. in the dihedral angle between two l.s. planes) are estimated using the full covariance matrix. The cell e.s.d.'s are taken into account individually in the estimation of e.s.d.'s in distances, angles and torsion angles; correlations between e.s.d.'s in cell parameters are only used when they are defined by crystal symmetry. An approximate (isotropic) treatment of cell e.s.d.'s is used for estimating e.s.d.'s involving l.s. planes.
Refinement. Refinement of *F*^2^ against ALL reflections. The weighted *R*-factor *wR* and goodness of fit *S* are based on *F*^2^, conventional *R*-factors *R* are based on *F*, with *F* set to zero for negative *F*^2^. The threshold expression of *F*^2^ > σ(*F*^2^) is used only for calculating *R*-factors(gt) *etc*. and is not relevant to the choice of reflections for refinement. *R*-factors based on *F*^2^ are statistically about twice as large as those based on *F*, and *R*- factors based on ALL data will be even larger.

### Fractional atomic coordinates and isotropic or equivalent isotropic displacement parameters (Å^2^)

**Table:**

	*x*	*y*	*z*	*U*_iso_*/*U*_eq_	
O1A	0.86360 (5)	0.58428 (18)	0.11715 (7)	0.0268 (3)	
O2A	0.87227 (5)	0.88589 (17)	0.10751 (6)	0.0222 (3)	
N1A	0.67465 (6)	0.8427 (2)	0.21685 (8)	0.0242 (3)	
N2A	0.05332 (5)	0.2784 (2)	0.45535 (7)	0.0180 (3)	
N3A	−0.04990 (6)	0.2128 (2)	0.55740 (7)	0.0199 (3)	
C1A	0.72048 (6)	0.8241 (2)	0.19910 (8)	0.0199 (3)	
C2A	0.74005 (7)	0.9733 (3)	0.17409 (9)	0.0235 (4)	
H2AA	0.7243	1.0919	0.1732	0.028*	
C3A	0.78230 (7)	0.9496 (3)	0.15049 (9)	0.0222 (4)	
H3AA	0.7954	1.0529	0.1342	0.027*	
C4A	0.80575 (6)	0.7776 (2)	0.15040 (8)	0.0182 (3)	
C5A	0.78674 (6)	0.6297 (2)	0.17669 (8)	0.0193 (3)	
H5AA	0.8025	0.5112	0.1776	0.023*	
C6A	0.74549 (6)	0.6517 (2)	0.20143 (8)	0.0195 (3)	
H6AA	0.7341	0.5495	0.2201	0.023*	
C7A	0.85012 (6)	0.7471 (2)	0.12315 (8)	0.0193 (3)	
C8A	0.07687 (6)	0.2119 (2)	0.52023 (8)	0.0169 (3)	
C9A	0.13552 (7)	0.1634 (2)	0.55063 (9)	0.0214 (3)	
H9AA	0.1579	0.1734	0.5253	0.026*	
C10A	0.16021 (7)	0.1021 (3)	0.61654 (9)	0.0239 (4)	
H10A	0.1998	0.0717	0.6368	0.029*	
C11A	0.12751 (7)	0.0835 (3)	0.65452 (9)	0.0226 (4)	
H11A	0.1452	0.0425	0.7004	0.027*	
C12A	0.07030 (7)	0.1245 (2)	0.62534 (8)	0.0193 (3)	
H12A	0.0483	0.1091	0.6509	0.023*	
C13A	0.04320 (6)	0.1901 (2)	0.55725 (8)	0.0162 (3)	
C14A	−0.01692 (6)	0.2343 (2)	0.52463 (8)	0.0166 (3)	
C15A	−0.04016 (6)	0.3026 (2)	0.45590 (8)	0.0167 (3)	
C16A	−0.09877 (6)	0.3509 (2)	0.41950 (8)	0.0195 (3)	
H16A	−0.1242	0.3381	0.4409	0.023*	
C17A	−0.11937 (7)	0.4155 (2)	0.35427 (8)	0.0217 (3)	
H17A	−0.1587	0.4480	0.3308	0.026*	
C18A	−0.08208 (7)	0.4338 (3)	0.32192 (8)	0.0229 (4)	
H18A	−0.0966	0.4777	0.2764	0.028*	
C19A	−0.02525 (7)	0.3891 (2)	0.35517 (8)	0.0208 (3)	
H19A	−0.0005	0.4028	0.3329	0.025*	
C20A	−0.00344 (6)	0.3226 (2)	0.42261 (8)	0.0171 (3)	
O1B	0.37845 (5)	0.68571 (17)	0.26050 (6)	0.0230 (3)	
O2B	0.36397 (5)	0.98772 (18)	0.25207 (6)	0.0261 (3)	
N1B	0.17296 (6)	0.7568 (2)	−0.03882 (8)	0.0229 (3)	
N2B	0.54393 (6)	0.2571 (2)	0.59970 (7)	0.0202 (3)	
N3B	0.44631 (6)	0.2602 (2)	0.39185 (7)	0.0220 (3)	
C1B	0.21918 (6)	0.7727 (2)	0.02383 (8)	0.0194 (3)	
C2B	0.23209 (7)	0.9435 (3)	0.05693 (9)	0.0245 (4)	
H2BA	0.2112	1.0494	0.0343	0.029*	
C3B	0.27492 (7)	0.9592 (3)	0.12218 (9)	0.0239 (4)	
H3BA	0.2830	1.0761	0.1437	0.029*	
C4B	0.30656 (6)	0.8068 (2)	0.15708 (8)	0.0190 (3)	
C5B	0.29449 (6)	0.6376 (2)	0.12345 (8)	0.0190 (3)	
H5BA	0.3158	0.5321	0.1460	0.023*	
C6B	0.25205 (7)	0.6210 (2)	0.05779 (9)	0.0203 (3)	
H6BA	0.2452	0.5051	0.0356	0.024*	
C7B	0.35265 (6)	0.8283 (2)	0.22785 (8)	0.0197 (3)	
C8B	0.48841 (7)	0.3151 (2)	0.57499 (8)	0.0198 (3)	
C9B	0.46617 (7)	0.3731 (3)	0.62140 (9)	0.0239 (4)	
H9BA	0.4899	0.3729	0.6689	0.029*	
C10B	0.41040 (7)	0.4296 (3)	0.59768 (9)	0.0260 (4)	
H10B	0.3956	0.4675	0.6291	0.031*	
C11B	0.37436 (7)	0.4325 (3)	0.52712 (9)	0.0227 (4)	
H11B	0.3356	0.4720	0.5113	0.027*	
C12B	0.39578 (7)	0.3779 (2)	0.48192 (9)	0.0225 (4)	
H12B	0.3716	0.3808	0.4345	0.027*	
C13B	0.45352 (6)	0.3169 (2)	0.50437 (8)	0.0193 (3)	
C14B	0.47737 (6)	0.2568 (2)	0.45815 (8)	0.0173 (3)	
C15B	0.53592 (6)	0.1946 (2)	0.48670 (8)	0.0161 (3)	
C16B	0.56309 (7)	0.1301 (2)	0.44572 (8)	0.0204 (3)	
H16B	0.5422	0.1278	0.3976	0.025*	
C17B	0.61859 (7)	0.0714 (3)	0.47412 (9)	0.0238 (4)	
H17B	0.6360	0.0285	0.4459	0.029*	
C18B	0.64998 (7)	0.0744 (3)	0.54509 (9)	0.0246 (4)	
H18B	0.6888	0.0344	0.5646	0.030*	
C19B	0.62536 (7)	0.1343 (2)	0.58664 (9)	0.0225 (4)	
H19B	0.6468	0.1340	0.6347	0.027*	
C20B	0.56781 (6)	0.1966 (2)	0.55777 (8)	0.0176 (3)	
O1W	0.43871 (6)	0.4726 (2)	0.20699 (7)	0.0283 (3)	
O2W	0.06211 (6)	0.5855 (2)	0.27425 (7)	0.0285 (3)	
O3W	0.46498 (6)	0.14630 (19)	0.27833 (6)	0.0279 (3)	
O4W	0.96560 (6)	0.42092 (19)	0.18638 (7)	0.0246 (3)	
H1NA	0.6681 (9)	0.745 (3)	0.2392 (11)	0.034 (6)*	
H2NA	0.6685 (9)	0.956 (4)	0.2310 (11)	0.043 (7)*	
H3NA	0.0766 (9)	0.302 (3)	0.4345 (10)	0.033 (5)*	
H4NA	−0.0364 (9)	0.170 (3)	0.6019 (11)	0.037 (6)*	
H5NA	−0.0885 (9)	0.234 (3)	0.5365 (10)	0.030 (5)*	
H1NB	0.1604 (9)	0.851 (3)	−0.0650 (11)	0.035 (6)*	
H2NB	0.1705 (9)	0.659 (3)	−0.0644 (11)	0.038 (6)*	
H3NB	0.5628 (10)	0.266 (3)	0.6495 (12)	0.051 (7)*	
H4NB	0.4051 (10)	0.296 (3)	0.3709 (11)	0.044 (6)*	
H5NB	0.4588 (9)	0.224 (3)	0.3608 (9)	0.041 (6)*	
H1W1	0.4462 (11)	0.373 (4)	0.2282 (13)	0.053 (8)*	
H2W1	0.4187 (11)	0.536 (4)	0.2230 (13)	0.055 (8)*	
H1W2	0.0841 (11)	0.525 (4)	0.3115 (14)	0.060 (8)*	
H2W2	0.0538 (11)	0.692 (4)	0.2861 (13)	0.055 (8)*	
H1W3	0.4345 (12)	0.077 (4)	0.2731 (14)	0.073 (9)*	
H2W3	0.4977 (11)	0.079 (4)	0.2807 (12)	0.052 (7)*	
H1W4	0.9960 (10)	0.486 (4)	0.2157 (12)	0.048 (7)*	
H2W4	0.9360 (11)	0.492 (4)	0.1677 (12)	0.050 (7)*	

### Atomic displacement parameters (Å^2^)

**Table:**

	*U*^11^	*U*^22^	*U*^33^	*U*^12^	*U*^13^	*U*^23^
O1A	0.0245 (6)	0.0221 (7)	0.0370 (7)	0.0003 (5)	0.0161 (5)	−0.0081 (6)
O2A	0.0229 (5)	0.0225 (7)	0.0252 (6)	−0.0014 (5)	0.0140 (5)	−0.0016 (5)
N1A	0.0249 (7)	0.0214 (9)	0.0320 (8)	−0.0004 (6)	0.0176 (6)	0.0004 (7)
N2A	0.0198 (6)	0.0182 (8)	0.0200 (7)	0.0004 (5)	0.0124 (6)	−0.0002 (5)
N3A	0.0181 (6)	0.0240 (8)	0.0192 (7)	−0.0002 (5)	0.0093 (6)	0.0018 (6)
C1A	0.0162 (7)	0.0235 (9)	0.0186 (8)	0.0002 (6)	0.0059 (6)	0.0001 (7)
C2A	0.0243 (8)	0.0178 (9)	0.0309 (9)	0.0042 (7)	0.0141 (7)	0.0020 (7)
C3A	0.0233 (8)	0.0199 (9)	0.0260 (9)	0.0005 (7)	0.0128 (7)	0.0033 (7)
C4A	0.0158 (7)	0.0203 (9)	0.0177 (8)	0.0000 (6)	0.0064 (6)	−0.0024 (6)
C5A	0.0163 (7)	0.0168 (9)	0.0207 (8)	0.0011 (6)	0.0036 (6)	−0.0022 (6)
C6A	0.0177 (7)	0.0176 (9)	0.0215 (8)	−0.0022 (6)	0.0064 (6)	0.0005 (7)
C7A	0.0157 (7)	0.0239 (10)	0.0160 (8)	0.0005 (6)	0.0045 (6)	−0.0035 (7)
C8A	0.0192 (7)	0.0134 (8)	0.0191 (8)	−0.0001 (6)	0.0091 (6)	−0.0016 (6)
C9A	0.0195 (7)	0.0208 (9)	0.0278 (9)	0.0012 (6)	0.0139 (7)	−0.0009 (7)
C10A	0.0184 (7)	0.0256 (10)	0.0270 (9)	0.0041 (7)	0.0089 (7)	−0.0003 (7)
C11A	0.0249 (8)	0.0223 (10)	0.0191 (8)	0.0031 (7)	0.0078 (7)	0.0012 (7)
C12A	0.0229 (7)	0.0180 (9)	0.0196 (8)	0.0001 (6)	0.0115 (6)	−0.0002 (6)
C13A	0.0188 (7)	0.0126 (8)	0.0188 (8)	−0.0004 (6)	0.0094 (6)	−0.0024 (6)
C14A	0.0200 (7)	0.0117 (8)	0.0204 (8)	−0.0020 (6)	0.0107 (6)	−0.0034 (6)
C15A	0.0199 (7)	0.0122 (8)	0.0189 (8)	−0.0007 (6)	0.0090 (6)	−0.0019 (6)
C16A	0.0191 (7)	0.0182 (9)	0.0230 (8)	−0.0012 (6)	0.0107 (6)	−0.0021 (7)
C17A	0.0208 (7)	0.0217 (10)	0.0207 (8)	0.0013 (6)	0.0069 (6)	−0.0012 (7)
C18A	0.0284 (8)	0.0227 (10)	0.0168 (8)	0.0008 (7)	0.0086 (7)	−0.0007 (7)
C19A	0.0258 (8)	0.0190 (9)	0.0211 (8)	−0.0009 (6)	0.0133 (7)	−0.0013 (7)
C20A	0.0203 (7)	0.0135 (8)	0.0193 (8)	−0.0016 (6)	0.0101 (6)	−0.0022 (6)
O1B	0.0241 (6)	0.0221 (7)	0.0224 (6)	0.0018 (5)	0.0094 (5)	0.0003 (5)
O2B	0.0246 (6)	0.0209 (7)	0.0302 (7)	−0.0014 (5)	0.0089 (5)	−0.0067 (5)
N1B	0.0218 (7)	0.0222 (9)	0.0235 (8)	−0.0007 (6)	0.0084 (6)	−0.0014 (7)
N2B	0.0231 (7)	0.0204 (8)	0.0160 (7)	0.0007 (6)	0.0072 (6)	0.0002 (6)
N3B	0.0188 (6)	0.0276 (9)	0.0182 (7)	0.0006 (6)	0.0063 (6)	−0.0009 (6)
C1B	0.0154 (7)	0.0233 (9)	0.0221 (8)	−0.0014 (6)	0.0105 (6)	−0.0002 (7)
C2B	0.0245 (8)	0.0179 (9)	0.0296 (9)	0.0019 (7)	0.0098 (7)	0.0005 (7)
C3B	0.0242 (8)	0.0178 (9)	0.0288 (9)	−0.0012 (7)	0.0103 (7)	−0.0046 (7)
C4B	0.0171 (7)	0.0194 (9)	0.0233 (8)	−0.0024 (6)	0.0112 (6)	−0.0019 (7)
C5B	0.0181 (7)	0.0180 (9)	0.0232 (8)	−0.0005 (6)	0.0107 (6)	0.0008 (7)
C6B	0.0208 (7)	0.0171 (9)	0.0281 (9)	−0.0035 (6)	0.0152 (7)	−0.0044 (7)
C7B	0.0175 (7)	0.0218 (9)	0.0238 (8)	−0.0009 (6)	0.0127 (6)	−0.0021 (7)
C8B	0.0241 (8)	0.0141 (9)	0.0229 (8)	−0.0028 (6)	0.0115 (7)	−0.0003 (6)
C9B	0.0275 (8)	0.0246 (10)	0.0199 (8)	−0.0012 (7)	0.0103 (7)	−0.0004 (7)
C10B	0.0274 (8)	0.0259 (10)	0.0285 (9)	0.0002 (7)	0.0154 (7)	−0.0017 (8)
C11B	0.0199 (7)	0.0226 (10)	0.0262 (9)	0.0025 (6)	0.0104 (7)	0.0013 (7)
C12B	0.0208 (7)	0.0214 (10)	0.0227 (8)	−0.0012 (6)	0.0067 (7)	0.0012 (7)
C13B	0.0169 (7)	0.0149 (9)	0.0258 (9)	−0.0012 (6)	0.0087 (6)	0.0009 (7)
C14B	0.0183 (7)	0.0131 (8)	0.0175 (8)	−0.0036 (6)	0.0045 (6)	0.0018 (6)
C15B	0.0168 (7)	0.0133 (8)	0.0173 (7)	−0.0008 (6)	0.0063 (6)	0.0022 (6)
C16B	0.0230 (7)	0.0202 (9)	0.0193 (8)	−0.0020 (6)	0.0099 (6)	0.0007 (7)
C17B	0.0228 (8)	0.0239 (10)	0.0294 (9)	0.0007 (7)	0.0156 (7)	0.0019 (7)
C18B	0.0154 (7)	0.0244 (10)	0.0322 (10)	0.0019 (6)	0.0082 (7)	0.0042 (8)
C19B	0.0197 (7)	0.0226 (10)	0.0189 (8)	−0.0009 (6)	0.0021 (6)	0.0027 (7)
C20B	0.0185 (7)	0.0147 (8)	0.0184 (8)	−0.0024 (6)	0.0065 (6)	0.0007 (6)
O1W	0.0313 (7)	0.0254 (8)	0.0297 (7)	0.0064 (6)	0.0143 (6)	0.0006 (6)
O2W	0.0336 (7)	0.0227 (8)	0.0252 (7)	0.0030 (6)	0.0085 (6)	0.0018 (6)
O3W	0.0266 (6)	0.0280 (8)	0.0281 (7)	−0.0028 (6)	0.0104 (5)	−0.0018 (6)
O4W	0.0265 (6)	0.0227 (7)	0.0247 (7)	0.0024 (5)	0.0110 (5)	−0.0016 (5)

### Geometric parameters (Å, º)

**Table:**

O1A—C7A	1.257 (2)	N1B—H2NB	0.89 (2)
O2A—C7A	1.273 (2)	N2B—C8B	1.363 (2)
N1A—C1A	1.390 (2)	N2B—C20B	1.365 (2)
N1A—H1NA	0.91 (2)	N2B—H3NB	0.98 (2)
N1A—H2NA	0.92 (3)	N3B—C14B	1.317 (2)
N2A—C8A	1.363 (2)	N3B—H4NB	0.99 (2)
N2A—C20A	1.365 (2)	N3B—H5NB	0.898 (9)
N2A—H3NA	0.90 (2)	C1B—C6B	1.396 (2)
N3A—C14A	1.3234 (19)	C1B—C2B	1.404 (2)
N3A—H4NA	0.93 (2)	C2B—C3B	1.383 (2)
N3A—H5NA	0.91 (2)	C2B—H2BA	0.9500
C1A—C2A	1.398 (2)	C3B—C4B	1.395 (2)
C1A—C6A	1.401 (2)	C3B—H3BA	0.9500
C2A—C3A	1.391 (2)	C4B—C5B	1.398 (2)
C2A—H2AA	0.9500	C4B—C7B	1.500 (2)
C3A—C4A	1.390 (2)	C5B—C6B	1.386 (2)
C3A—H3AA	0.9500	C5B—H5BA	0.9500
C4A—C5A	1.398 (2)	C6B—H6BA	0.9500
C4A—C7A	1.504 (2)	C8B—C13B	1.410 (2)
C5A—C6A	1.383 (2)	C8B—C9B	1.413 (2)
C5A—H5AA	0.9500	C9B—C10B	1.366 (2)
C6A—H6AA	0.9500	C9B—H9BA	0.9500
C8A—C9A	1.412 (2)	C10B—C11B	1.415 (2)
C8A—C13A	1.414 (2)	C10B—H10B	0.9500
C9A—C10A	1.370 (2)	C11B—C12B	1.368 (2)
C9A—H9AA	0.9500	C11B—H11B	0.9500
C10A—C11A	1.407 (2)	C12B—C13B	1.424 (2)
C10A—H10A	0.9500	C12B—H12B	0.9500
C11A—C12A	1.367 (2)	C13B—C14B	1.443 (2)
C11A—H11A	0.9500	C14B—C15B	1.438 (2)
C12A—C13A	1.422 (2)	C15B—C20B	1.407 (2)
C12A—H12A	0.9500	C15B—C16B	1.420 (2)
C13A—C14A	1.439 (2)	C16B—C17B	1.362 (2)
C14A—C15A	1.439 (2)	C16B—H16B	0.9500
C15A—C20A	1.415 (2)	C17B—C18B	1.403 (2)
C15A—C16A	1.419 (2)	C17B—H17B	0.9500
C16A—C17A	1.366 (2)	C18B—C19B	1.370 (2)
C16A—H16A	0.9500	C18B—H18B	0.9500
C17A—C18A	1.411 (2)	C19B—C20B	1.416 (2)
C17A—H17A	0.9500	C19B—H19B	0.9500
C18A—C19A	1.368 (2)	O1W—H1W1	0.84 (3)
C18A—H18A	0.9500	O1W—H2W1	0.87 (3)
C19A—C20A	1.411 (2)	O2W—H1W2	0.88 (3)
C19A—H19A	0.9500	O2W—H2W2	0.87 (3)
O1B—C7B	1.273 (2)	O3W—H1W3	0.90 (3)
O2B—C7B	1.256 (2)	O3W—H2W3	0.96 (3)
N1B—C1B	1.381 (2)	O4W—H1W4	0.91 (3)
N1B—H1NB	0.86 (2)	O4W—H2W4	0.87 (3)
			
C1A—N1A—H1NA	115.8 (13)	H1NB—N1B—H2NB	109 (2)
C1A—N1A—H2NA	117.7 (14)	C8B—N2B—C20B	121.86 (14)
H1NA—N1A—H2NA	115.3 (19)	C8B—N2B—H3NB	111.3 (13)
C8A—N2A—C20A	122.22 (13)	C20B—N2B—H3NB	126.9 (14)
C8A—N2A—H3NA	118.9 (13)	C14B—N3B—H4NB	122.9 (13)
C20A—N2A—H3NA	118.7 (13)	C14B—N3B—H5NB	124.5 (14)
C14A—N3A—H4NA	123.4 (13)	H4NB—N3B—H5NB	112.5 (19)
C14A—N3A—H5NA	121.4 (13)	N1B—C1B—C6B	122.06 (16)
H4NA—N3A—H5NA	115.1 (18)	N1B—C1B—C2B	119.75 (16)
N1A—C1A—C2A	121.01 (16)	C6B—C1B—C2B	118.08 (15)
N1A—C1A—C6A	120.43 (16)	C3B—C2B—C1B	120.71 (17)
C2A—C1A—C6A	118.41 (14)	C3B—C2B—H2BA	119.6
C3A—C2A—C1A	120.60 (16)	C1B—C2B—H2BA	119.6
C3A—C2A—H2AA	119.7	C2B—C3B—C4B	121.29 (17)
C1A—C2A—H2AA	119.7	C2B—C3B—H3BA	119.4
C4A—C3A—C2A	121.16 (16)	C4B—C3B—H3BA	119.4
C4A—C3A—H3AA	119.4	C3B—C4B—C5B	117.89 (15)
C2A—C3A—H3AA	119.4	C3B—C4B—C7B	120.02 (16)
C3A—C4A—C5A	117.87 (14)	C5B—C4B—C7B	122.08 (15)
C3A—C4A—C7A	122.25 (15)	C6B—C5B—C4B	121.15 (16)
C5A—C4A—C7A	119.88 (15)	C6B—C5B—H5BA	119.4
C6A—C5A—C4A	121.61 (16)	C4B—C5B—H5BA	119.4
C6A—C5A—H5AA	119.2	C5B—C6B—C1B	120.81 (16)
C4A—C5A—H5AA	119.2	C5B—C6B—H6BA	119.6
C5A—C6A—C1A	120.28 (16)	C1B—C6B—H6BA	119.6
C5A—C6A—H6AA	119.9	O2B—C7B—O1B	123.11 (15)
C1A—C6A—H6AA	119.9	O2B—C7B—C4B	117.87 (15)
O1A—C7A—O2A	123.20 (14)	O1B—C7B—C4B	119.02 (15)
O1A—C7A—C4A	117.83 (15)	N2B—C8B—C13B	120.76 (14)
O2A—C7A—C4A	118.97 (15)	N2B—C8B—C9B	118.87 (15)
N2A—C8A—C9A	119.55 (14)	C13B—C8B—C9B	120.36 (15)
N2A—C8A—C13A	120.78 (13)	C10B—C9B—C8B	119.75 (16)
C9A—C8A—C13A	119.67 (14)	C10B—C9B—H9BA	120.1
C10A—C9A—C8A	120.19 (14)	C8B—C9B—H9BA	120.1
C10A—C9A—H9AA	119.9	C9B—C10B—C11B	121.14 (16)
C8A—C9A—H9AA	119.9	C9B—C10B—H10B	119.4
C9A—C10A—C11A	120.74 (15)	C11B—C10B—H10B	119.4
C9A—C10A—H10A	119.6	C12B—C11B—C10B	119.38 (15)
C11A—C10A—H10A	119.6	C12B—C11B—H11B	120.3
C12A—C11A—C10A	119.94 (15)	C10B—C11B—H11B	120.3
C12A—C11A—H11A	120.0	C11B—C12B—C13B	121.41 (16)
C10A—C11A—H11A	120.0	C11B—C12B—H12B	119.3
C11A—C12A—C13A	121.01 (14)	C13B—C12B—H12B	119.3
C11A—C12A—H12A	119.5	C8B—C13B—C12B	117.95 (15)
C13A—C12A—H12A	119.5	C8B—C13B—C14B	119.16 (14)
C8A—C13A—C12A	118.41 (14)	C12B—C13B—C14B	122.88 (15)
C8A—C13A—C14A	118.87 (14)	N3B—C14B—C15B	121.28 (15)
C12A—C13A—C14A	122.72 (13)	N3B—C14B—C13B	120.70 (14)
N3A—C14A—C15A	120.96 (14)	C15B—C14B—C13B	118.02 (14)
N3A—C14A—C13A	120.49 (14)	C20B—C15B—C16B	118.28 (14)
C15A—C14A—C13A	118.55 (13)	C20B—C15B—C14B	119.32 (14)
C20A—C15A—C16A	118.08 (14)	C16B—C15B—C14B	122.40 (14)
C20A—C15A—C14A	119.11 (13)	C17B—C16B—C15B	121.18 (15)
C16A—C15A—C14A	122.81 (14)	C17B—C16B—H16B	119.4
C17A—C16A—C15A	121.24 (14)	C15B—C16B—H16B	119.4
C17A—C16A—H16A	119.4	C16B—C17B—C18B	119.99 (15)
C15A—C16A—H16A	119.4	C16B—C17B—H17B	120.0
C16A—C17A—C18A	119.80 (15)	C18B—C17B—H17B	120.0
C16A—C17A—H17A	120.1	C19B—C18B—C17B	120.81 (15)
C18A—C17A—H17A	120.1	C19B—C18B—H18B	119.6
C19A—C18A—C17A	120.89 (15)	C17B—C18B—H18B	119.6
C19A—C18A—H18A	119.6	C18B—C19B—C20B	119.78 (15)
C17A—C18A—H18A	119.6	C18B—C19B—H19B	120.1
C18A—C19A—C20A	119.83 (15)	C20B—C19B—H19B	120.1
C18A—C19A—H19A	120.1	N2B—C20B—C15B	120.86 (14)
C20A—C19A—H19A	120.1	N2B—C20B—C19B	119.18 (14)
N2A—C20A—C19A	119.40 (13)	C15B—C20B—C19B	119.96 (14)
N2A—C20A—C15A	120.45 (14)	H1W1—O1W—H2W1	107 (2)
C19A—C20A—C15A	120.15 (14)	H1W2—O2W—H2W2	108 (2)
C1B—N1B—H1NB	120.6 (15)	H1W3—O3W—H2W3	114 (2)
C1B—N1B—H2NB	119.1 (14)	H1W4—O4W—H2W4	110 (2)
			
N1A—C1A—C2A—C3A	−173.95 (16)	N1B—C1B—C2B—C3B	174.26 (15)
C6A—C1A—C2A—C3A	1.6 (2)	C6B—C1B—C2B—C3B	−2.1 (2)
C1A—C2A—C3A—C4A	0.7 (3)	C1B—C2B—C3B—C4B	0.0 (3)
C2A—C3A—C4A—C5A	−1.9 (2)	C2B—C3B—C4B—C5B	1.4 (2)
C2A—C3A—C4A—C7A	178.03 (15)	C2B—C3B—C4B—C7B	−179.87 (15)
C3A—C4A—C5A—C6A	0.7 (2)	C3B—C4B—C5B—C6B	−0.7 (2)
C7A—C4A—C5A—C6A	−179.19 (14)	C7B—C4B—C5B—C6B	−179.40 (14)
C4A—C5A—C6A—C1A	1.6 (2)	C4B—C5B—C6B—C1B	−1.4 (2)
N1A—C1A—C6A—C5A	172.84 (15)	N1B—C1B—C6B—C5B	−173.47 (14)
C2A—C1A—C6A—C5A	−2.8 (2)	C2B—C1B—C6B—C5B	2.8 (2)
C3A—C4A—C7A—O1A	−171.22 (15)	C3B—C4B—C7B—O2B	−4.6 (2)
C5A—C4A—C7A—O1A	8.7 (2)	C5B—C4B—C7B—O2B	174.05 (14)
C3A—C4A—C7A—O2A	9.0 (2)	C3B—C4B—C7B—O1B	175.43 (14)
C5A—C4A—C7A—O2A	−171.12 (14)	C5B—C4B—C7B—O1B	−5.9 (2)
C20A—N2A—C8A—C9A	178.09 (16)	C20B—N2B—C8B—C13B	−0.8 (2)
C20A—N2A—C8A—C13A	−1.6 (2)	C20B—N2B—C8B—C9B	178.96 (16)
N2A—C8A—C9A—C10A	177.90 (16)	N2B—C8B—C9B—C10B	−179.17 (17)
C13A—C8A—C9A—C10A	−2.5 (3)	C13B—C8B—C9B—C10B	0.6 (3)
C8A—C9A—C10A—C11A	1.1 (3)	C8B—C9B—C10B—C11B	−0.5 (3)
C9A—C10A—C11A—C12A	0.9 (3)	C9B—C10B—C11B—C12B	0.0 (3)
C10A—C11A—C12A—C13A	−1.4 (3)	C10B—C11B—C12B—C13B	0.5 (3)
N2A—C8A—C13A—C12A	−178.44 (15)	N2B—C8B—C13B—C12B	179.58 (15)
C9A—C8A—C13A—C12A	1.9 (2)	C9B—C8B—C13B—C12B	−0.2 (2)
N2A—C8A—C13A—C14A	1.7 (2)	N2B—C8B—C13B—C14B	−0.2 (2)
C9A—C8A—C13A—C14A	−177.91 (15)	C9B—C8B—C13B—C14B	−179.97 (16)
C11A—C12A—C13A—C8A	0.0 (2)	C11B—C12B—C13B—C8B	−0.3 (3)
C11A—C12A—C13A—C14A	179.83 (16)	C11B—C12B—C13B—C14B	179.42 (17)
C8A—C13A—C14A—N3A	178.94 (16)	C8B—C13B—C14B—N3B	−178.63 (16)
C12A—C13A—C14A—N3A	−0.9 (2)	C12B—C13B—C14B—N3B	1.6 (3)
C8A—C13A—C14A—C15A	−1.2 (2)	C8B—C13B—C14B—C15B	1.0 (2)
C12A—C13A—C14A—C15A	178.99 (15)	C12B—C13B—C14B—C15B	−178.80 (15)
N3A—C14A—C15A—C20A	−179.66 (16)	N3B—C14B—C15B—C20B	178.81 (16)
C13A—C14A—C15A—C20A	0.5 (2)	C13B—C14B—C15B—C20B	−0.8 (2)
N3A—C14A—C15A—C16A	0.2 (2)	N3B—C14B—C15B—C16B	−1.4 (2)
C13A—C14A—C15A—C16A	−179.69 (15)	C13B—C14B—C15B—C16B	179.00 (15)
C20A—C15A—C16A—C17A	−0.2 (2)	C20B—C15B—C16B—C17B	0.0 (2)
C14A—C15A—C16A—C17A	179.94 (16)	C14B—C15B—C16B—C17B	−179.77 (16)
C15A—C16A—C17A—C18A	0.4 (3)	C15B—C16B—C17B—C18B	−0.1 (3)
C16A—C17A—C18A—C19A	−0.6 (3)	C16B—C17B—C18B—C19B	0.6 (3)
C17A—C18A—C19A—C20A	0.4 (3)	C17B—C18B—C19B—C20B	−0.9 (3)
C8A—N2A—C20A—C19A	−179.39 (15)	C8B—N2B—C20B—C15B	1.0 (2)
C8A—N2A—C20A—C15A	0.8 (2)	C8B—N2B—C20B—C19B	−178.62 (16)
C18A—C19A—C20A—N2A	179.99 (16)	C16B—C15B—C20B—N2B	−179.97 (15)
C18A—C19A—C20A—C15A	−0.2 (2)	C14B—C15B—C20B—N2B	−0.2 (2)
C16A—C15A—C20A—N2A	179.90 (15)	C16B—C15B—C20B—C19B	−0.3 (2)
C14A—C15A—C20A—N2A	−0.3 (2)	C14B—C15B—C20B—C19B	179.44 (15)
C16A—C15A—C20A—C19A	0.1 (2)	C18B—C19B—C20B—N2B	−179.56 (16)
C14A—C15A—C20A—C19A	179.93 (15)	C18B—C19B—C20B—C15B	0.8 (3)

### Hydrogen-bond geometry (Å, º)

*Cg*13 is the centroid of the C1A–C6A ring.

**Table:**

*D*—H···*A*	*D*—H	H···*A*	*D*···*A*	*D*—H···*A*
O1*W*—H1*W*1···O3*W*	0.84 (3)	1.92 (3)	2.758 (2)	179 (5)
O1*W*—H2*W*1···O1*B*	0.86 (3)	1.90 (3)	2.767 (2)	177 (2)
O2*W*—H1*W*2···O2*A*^i^	0.88 (3)	1.92 (3)	2.8066 (19)	176 (3)
O3*W*—H2*W*3···O1*W*^i^	0.96 (3)	1.72 (3)	2.676 (2)	174 (2)
O3*W*—H1*W*3···O2*B*^ii^	0.90 (3)	1.79 (3)	2.674 (2)	166 (3)
O4*W*—H2*W*4···O1*A*	0.87 (3)	1.84 (3)	2.688 (2)	164 (3)
N1*A*—H1*NA*···O2*B*^i^	0.92 (2)	2.09 (2)	2.946 (2)	156.0 (19)
N1*A*—H2*NA*···O1*B*^iii^	0.92 (3)	2.12 (3)	2.983 (2)	158 (2)
N2*A*—H3*NA*···O2*A*^i^	0.90 (2)	1.98 (2)	2.882 (2)	173 (2)
N2*B*—H3*NB*···O1*B*^iv^	0.98 (2)	1.93 (2)	2.8695 (18)	160 (2)
N3*A*—H4*NA*···O4*W*^v^	0.93 (2)	1.93 (2)	2.8227 (19)	161.6 (18)
N3*A*—H5*NA*···N1*B*^vi^	0.91 (2)	2.20 (2)	3.031 (2)	151.4 (18)
N3*B*—H5*NB*···O3*W*	0.90 (2)	1.94 (2)	2.819 (2)	165 (2)
N3*B*—H4*NB*···N1*A*^i^	1.00 (3)	2.16 (2)	3.067 (2)	151.3 (19)
C12*A*—H12*A*···O4*W*^v^	0.95	2.55	3.4756 (19)	164
C16*B*—H16*B*···O3*W*	0.95	2.51	3.438 (2)	164
C11*A*—H11*A*···*Cg*13^iv^	0.95	2.95	3.6659 (19)	133

Symmetry codes: (i) −*x*+1, *y*−1/2, −*z*+1/2; (ii) *x*, *y*−1, *z*; (iii) −*x*+1, *y*+1/2, −*z*+1/2; (iv) −*x*+1, −*y*+1, −*z*+1; (v) *x*−1, −*y*+1/2, *z*+1/2; (vi) −*x*, *y*−1/2, −*z*+1/2.
